# ACC Deaminase Produced by PGPR Mitigates the Adverse Effect of Osmotic and Salinity Stresses in *Pisum sativum* through Modulating the Antioxidants Activities

**DOI:** 10.3390/plants11243419

**Published:** 2022-12-07

**Authors:** Anmol Gupta, Smita Rai, Ambreen Bano, Swati Sharma, Manoj Kumar, Reem Binsuwaidan, Mohammad Suhail Khan, Tarun Kumar Upadhyay, Nawaf Alshammari, Mohd Saeed, Neelam Pathak

**Affiliations:** 1 IIRC-3, Plant-Microbe Interaction and Molecular Immunology Laboratory, Department of Biosciences, Faculty of Science, Integral University, Lucknow 226026, India; 2 CSIR—National Botanical Research Institute, Rana Pratap Marg, Lucknow 226001, India; 3 Department of Pharmaceutical Sciences, College of Pharmacy, Princess Nourah Bint Abdulrahman University, Riyadh 11671, Saudi Arabia; 4 Department of Public Health, College of Applied Medical Sciences, Khamis Mushait Campus, King Khalid University, Abha 62521, Saudi Arabia; 5 Department of Biotechnology, Parul Institute of Applied Sciences and Centre for Research for Development, Parul University, Vadodara 391760, India; 6 Department of Biology, College of Sciences, University of Hail, Hail 55476, Saudi Arabia; 7 Department of Biochemistry, Dr. Ram Manohar Lohia Avadh University, Ayodhya 224001, India

**Keywords:** salinity stress, ACC deaminase, PGPR, *Pisum sativum*, ROS, antioxidant enzymes

## Abstract

Salinity-induced ethylene production and reactive oxygen species (ROS) inhibit agricultural productivity. The plant synthesizes ethylene directly from aminocyclopropane-1-carboxylic acid (ACC). By using ACC as a nitrogen source, bacteria with ACC deaminase (ACCD) inhibit the overproduction of ethylene, thereby maintaining the ROS. The present study investigated the ACCD activity of previously identified rhizobacterial strains in Dworkin and Foster (DF) minimal salt media supplemented with 5 mM ACC (as N-source). Bacterial isolates GKP KS2_7 (*Pseudomonas aeruginosa*) and MBD 133 (*Bacillus subtilis*) could degrade ACC into α-ketobutyrate, exhibiting ACCD activity producing more than ~257 nmol of α-ketobutyrate mg protein^−1^ h^−1^, and were evaluated for other plant growth-promoting (PGP) traits including indole acetic acid production (>63 µg/mL), phosphate solubilization (>86 µg mL^−1^), siderophore (>20%) ammonia and exopolysaccharide production. Furthermore, Fourier Transform Infrared analysis also demonstrated α-ketobutyrate liberation from ACC deamination in DF minimal salt media, thereby confirming the ACCD activity. These isolates also showed enhanced tolerance to salinity stress of 3% *w/v* NaCl in vitro, in addition to facilitating multifarious PGP activities. Seed bacterization by these ACCD-producing bacterial isolates (GKP KS2_7 and MBD 133) revealed a significant decline in stress-stimulated ethylene levels and its associated growth inhibition during seedling germination. They also mitigated the negative effects of salt stress and increased the root-shoot length, fresh and dry weight of root and shoot, root-shoot biomass, total sugar, protein, reducing sugar, chlorophyll content, and antioxidants enzymes in *Pisum sativum*. As a result, these strains (GKP KS2_7 and MBD 133) might be applied as biofertilizers to counteract the negative effects of soil salinity.

## 1. Introduction

In the current global food crisis, developing a sustainable agricultural system is one of the major challenges. Abiotic stresses can severely affect crop yield and agricultural land and alone can reduce the average crop yield by up to 50% [1]. Out of these abiotic stresses, salinity stress is critically important. Salinity stress affects up to 6% of the total land area and 30% of the irrigated land area globally [2], resulting in huge economic losses [3]. Salinity causes various detrimental effects on plants, for instance, reduction in the photosynthetic pigments (chlorophylls and carotenoids), stomatal closure, protein synthesis, respiration, as well as changes in their morphological and anatomical features, lipid metabolism, energy transformation, and ionic imbalance that is ultimately inhibiting the growth and development of plants [4,5].

The secondary effects of salinity stress include osmotic and oxidative stress caused due to over-production of reactive oxygen species (ROS), thereby causing electrolyte damage, membrane lipid peroxidation, damage to the cellular components such as nucleic acids, membrane proteins, and lipids, and leading to metabolic dysfunction [6,7]. It has been observed that the ethylene level is elevated in seeds under exposure to salinity stress [8]. Seed germination and root development are both hampered by excessive ethylene [9,10], conferring negative effects on plants. Under numerous environmental conditions, different bacteria exhibit diverse levels of enzyme activity. However, bacteria that have the enzyme ACC (1-aminocyclopropane-1-carboxylate) deaminase (ACCD) may hydrolyze ACC, the precursor of ethylene, enabling surplus ethylene to prevent and protect plants from these inhibitory effects [11,12]. When inoculated, several ACCD-positive bacterial isolates have also been observed to cause an upsurge in fresh and dry biomass, photosynthetic pigments, several flowers, and buds in plants as compared to non-inoculated [12,13,14], thereby acting as plant growth-promoting rhizobacteria (PGPR). For example, acdS^+^ isolate *Achromobacter piechaudii* strain ARV8 was found to be effective in fostering tomato plant growth under induced salinity stress [15]. These ACCD-producing acdS gene-containing bacterial isolates can confer an increase in the activities of antioxidant enzymes such as SOD (superoxide dismutase) and CAT (catalase), APX (ascorbate peroxidase), POD (peroxidase), LPX (lipid peroxidase) and GPX (glutathione peroxidase) in plants that play a crucial role in protecting cells from the detrimental effects of ROS in salt stress conditions [12,16,17].

ACCD-producing PGPR, *Pseudomonas putida,* and *Bacillus amyloliquefaciens* showed tolerance to pesticides at concentrations such as Imidacloprid (3.27%), Carbendazim (0.512%) and Glyphosate (3.27%) in chickpea rhizosphere through modulating antioxidant enzymatic activities as well as by producing IAA, exopolysaccharides (EPS), biofilm, siderophores, and phosphate (P) solubilization [18]. Additionally, Gupta et al. [19] also investigated the effect of the ACCD salt tolerant-PGPR traits on various crops. Several other researchers also studied the effects of acdS^+^ strains on plant growth under abiotic stresses, including salinity stress [12,16,20,21]. *Bacillus subtilis* can solubilize soil P, produces siderophores, and enhance nitrogen fixation, which promotes its growth and suppresses pathogens’ growth. It enhances stress tolerance in their plant hosts by inducing the expression of stress-response genes, phytohormones, and stress-related metabolites [22]. Similarly, the *Pseudomonas aeruginosa* strain FG106 strain exhibited multiple PGP traits and showed a high rate of inhibition of growth and pathogenicity of tested phytopathogens in vitro and in vivo [23].

The pea (*Pisum sativum*) plant is very vulnerable to salinity stress in its early stages of growth, lowering its productivity and output [12,24,25,26,27]. Plants’ morphological and physiological responses are adversely affected by salinity, thereby affecting their metabolism [12,28,29]. In addition to reduced growth, salinity reduces germination percentage, leaf length, shoot-root freshness, and dry weight [12,16,30], ultimately, farm yield and productivity [31]. 

In our research, two rhizospheric bacteria *Pseudomonas aeruginosa* (GKP KS2_7) and *Bacillus subtilis* (MBD 133), were examined for their salinity (NaCl) tolerance, PGP abilities, and potential to reduce salt stress in pea plants. Henceforth, the objective of the present study aimed to reveal the beneficiary effect of ACCD-producing bacteria on *P. sativum* by bio-priming the seeds with individual isolates under normal and salt stress conditions, thereby examining the physiological as well as biochemical parameters. The in vitro and pot experiments showed that *P. aeruginosa* (GKP KS2 7) and *B. subtilis* (MBD 133) could promote plant growth in salt-stressed soil by stimulating biochemical mechanisms in salt-stress lands.

## 2. Results

### 2.1. Bacterial Strains and Their Plant Growth-Promoting Traits

*Pseudomonas aeruginosa* (GKP KS2_7) and *Bacillus subtilis* (MBD 133) strains were screened positive for IAA production, P-solubilization, ACCD activity, siderophore production, NH_3_ production, and EPS production (Table 1). When considering their ability to promote pea growth under salinity stress conditions, both bacterial isolates were therefore assumed to be plant growth-promoting rhizobacteria.

### 2.2. Ninhydrin ACC Assay for Selected Bacteria

The bacterial isolates GKP KS2_7 and MBD 133 that utilized ACC in DF-media showed an ACCD activity, indicating that the screening process works effectively (data not shown). In this study, colorimetric ninhydrin assay is used to determine the consumption of the substrate ACC (the sole N-source) during bacterial growth in a medium as ninhydrin-ACC assay results showed a direct correlation between ACC (substrate) utilization by bacteria and ACC deaminase activity. ACC-using bacterial strains used up, or nearly consumed, the initial 0.015 mmol l^−1^ ACC after the incubation of 24 h, which resulted in a noticeable decrease in the final color depth compared to the DF-ACC medium that was not inoculated. Among them, the ACC consumption activity ranging from 0.597 ± 0.002 GKP KS2_7 to 0.220 ± 0.002 MBD 133 (0 h) and 0.341 ± 0.001 GKP KS2_7 to 0.030 ± 0.001 MBD 133 (24 h) nmol α-ketobutyrate per mg protein per h revealed differences in ACC utilization (Table 2).

### 2.3. ACC Deaminase Activity and Confirmation of Pseudomonas and Bacillus Species Using FTIR

**Figure 1:**
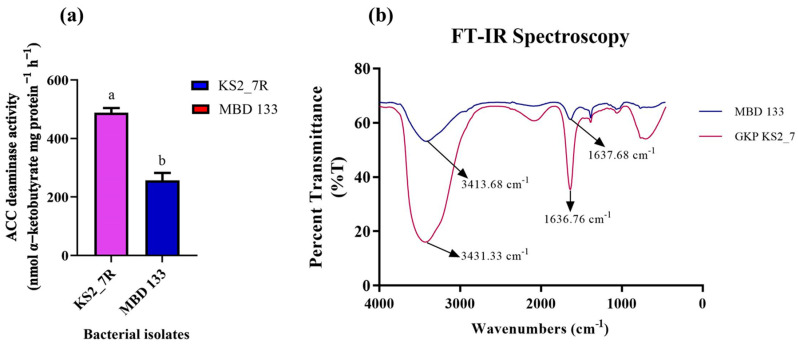
(**a**) Quantitative estimation of ACC deaminase activity; (**b**) FTIR spectra of α-ketobutyrate in the strains. GKP KS2_7 (*Pseudomonas aeruginosa*) and MBD 133 (*Bacillus subtilis*). Values are the mean of three replicates. Mean ± standard error (SE) is indicated by error bars. Statistical significance is indicated by differences in letters between treatments (unpaired *t*-test, two-tailed at *p* < 0.05). A different letter is an indication of a significant difference, indicating the significance between them.

Both the isolates showed variation in ACCD activity in the range of 257–489 nmol α-ketobutyrate per mg of cellular protein per hour. The highest ACCD activity was exhibited by *P. aeruginosa* KS2_7 (489 nmol α-ketobutyrate mg protein^−1^ h^−1^) followed by *B. subtilis* MBD 133 (257 nmol α-ketobutyrate mg protein^−1^ h^−1^) (Figure 1a). The highest enzymatic activity of ACCD produced by both the strains, i.e., conversion of N source ACC into α-ketobutyrate, was further confirmed by FTIR spectrum analysis (Figure 1b), which displays peaks at 3413 and 1637 cm^−1^ in MBD 133 and 3431 and 1636 in GKP KS2_7, validating the existence of a ketonic group and amino functional group, respectively identified as α-ketobutyrate as per Sarkar et al. [32]. Alpha ketobutyrate FTIR spectra showed strong evidence of functional amino groups (−NH_2_) at peak 3413 to 3431 cm^−1^ in the expected keto ester. In order to determine the presence of a keto functional group (−C=O) attached to an organic fragment of molecular structure such as an ester, there was a terminal alkene and a peak at 1637 cm^−1^. All these findings provide evidence for the structure and nature of organic molecules (Figure 1b). An FTIR analysis of the GKP KS2_7 and MBD 133 reaction mixture showed similarities in peak shape and confirmed that the product was α-ketobutyrate (Figure 1b).

### 2.4. NaCl Maximum Tolerance Level (MTL)

**Figure 2:**
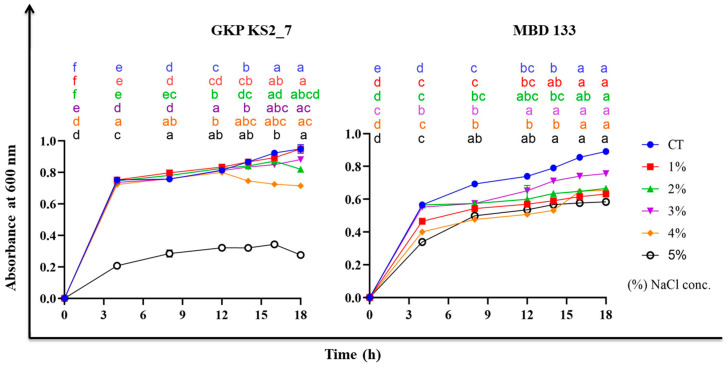
Growth curve analysis of bacterial strains under different salt concentrations. CT—no salt inoculation; GKP KS2_7 (*Pseudomonas aeruginosa*) and MBD 133 (*Bacillus subtilis*)—Bacterial treatments; 1-, 2-, 3-, 4-, and 5%—different NaCl concentration. Values are the mean of three replicates. Mean ± standard error (SE) is indicated by error bars. Statistical significance is indicated by differences in letters between treatments (Two-way ANOVA, Tukey’s multiple comparison test *p* < 0.05). A different letter is an indication of a significant difference, indicating the significance between them.

In our investigation, 3% NaCl (*w/v*) didn’t affect the bacterial count of *Bacillus* and *Pseudomonas* isolates’. However, the optical density (O.D.) value decreased with increasing salt concentrations, resulting in significant inhibition of bacterial growth (Figure 2) and on inoculation of 5% NaCl in nutrient broth (NB) both the bacterial isolates reduces the bacterial growth. Hence, the maximum salt tolerance of these isolates is therefore 3% NaCl (*w/v*).

### 2.5. Effect of PGPR Strains on Seedlings Parameters under Salinity Stress Conditions

Results showed that salinity adversely affected the seedlings of *P. sativum*. Both the salt-tolerant (ST) bacterial isolates (*P. aeruginosa* and *B. subtilis*) enhanced the seedling parameters under 1% induced salinity stress (Table 3). Seeds treated with *Pseudomonas* sp. GKP KS2_7 and *Bacillus* sp. MBD 133 enhanced the germination percent (G%) by 91 and 67% over the negative control. However, these isolates were further used to evaluate their effect on other seedling parameters (Table 3). The effects of the bacterial strains on seed G%, GI, MGT, TGP, CVG, GRI, and seedling VI in pea seedlings are summarized in (Table 3).

### 2.6. Morphological Parameters of Pisum sativum under Salinity Stress and in the Presence of PGPR

**Figure 3:**
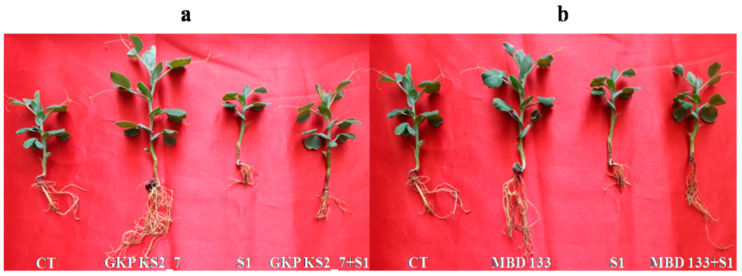
Effect of acdS^+^ PGPR strains on *Pisum sativum* growth under normal and 1% NaCl induced salinity stress after 25 DAS. (**a**) CT—control without bacterial inoculants and salt; GKP KS2_7—*Pseudomonas aeruginosa*; Bacterial treated—GKP KS2_7+S1; S1—1% NaCl; (**b**) CT—control without bacterial inoculants and salt; MBD 133—*Bacillus subtilis*; Bacterial treated; MBD 133+S1—PGPR+NaCl; S1—1% NaCl.

The *P. sativum* plants were cultivated in pots under greenhouse (GH) conditions to assess the effects of these ST PGPR isolates—GKP KS2 7 (*P. aeruginosa*) and MBD 133 (*B. subtilis*). Plants treated with only NaCl experienced retarded growth compared to those treated with PGPR-inoculated NaCl (Figure 3a,b, Table 4). The advantages of these acdS^+^ PGPR under saline and non-saline environments for *P. sativum* are explored in (pot trials) GH experiments (Figure 3). After planting, a biomass measurement was performed up to 25 days after sowing (DAS). Plants were harvested for measuring various morphological characteristics such as root and shoot length, and fresh and dry weight of root-shoot (Table 4).

The results showed that 1% NaCl adversely affected plant growth parameters, resulting in a reduction in fresh weight (FW), dry weight (DW), stem length (SL), and root length (RL) (Figure 3a,b; Table 4). On inoculation, acdS^+^ GKP KS2_7 and MBD 133 isolates displayed significantly different growth characteristics than non-inoculated plants (Figure 3a,b). Thus, salinity stress may negatively affect plant growth, but inoculation of these acdS^+^ isolates may counteract it.

*Pisum sativum* growth and biomass were affected by NaCl stress, indicating a marked reduction in RL, SL, FW, and DW as compared to non-stressed controls (Table 4). In contrast with non-inoculated plants, the application of ST GKP KS2_7 (*P. aeruginosa*) and MBD 133 (*B. subtilis*) acdS^+^ PGPR strains significantly improved root and shoot growth in *P. sativum* (*p* < 0.05). The pots trial results indicate that plants inoculated with acdS^+^ strains showed better results and could tolerate NaCl-induced stress better compared to the control plants. In addition, both isolates were found to promote all the physiological parameters under salinity stress conditions (Table 4). Moreover, on inoculation, *Pseudomonas* sp. (GKP KS2_7) exhibited the highest fresh weight and improved the length of roots and shoots in comparison to *Bacillus* sp. (MBD 133) under salinity stress conditions (Table 4).

### 2.7. Effect on Biochemical Parameters

#### 2.7.1. Total Soluble Sugar (TSS), Reducing Sugar (RS), and Protein

**Figure 4:**
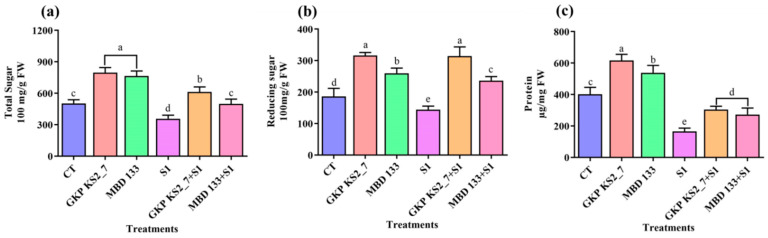
Effect of acdS^+^ bacterial isolates on biochemical parameters under induced NaCl stress in pea. (**a**) Total sugar content; (**b**) Total reducing sugar content; and (**c**) Total protein content. Values are the mean of five replicates mean ± standard error (SE). Normal soil: CT—Control; GKP KS2_7—*Pseudomonas aeruginosa*; MBD 133—*Bacillus subtilis*: Salt stressed: S1—1% NaCl; GKP KS2_7+S1 and MBD 133+S1—PGPR+NaCl. Statistical significance is indicated by differences in letters between treatments (One-way ANOVA, Tukey’s multiple range test *p* < 0.05). Similar letters represent no significance, while a different letter is indicative of a significant difference indicating the significance between them.

The biochemical parameters such as TSS, RS, and protein content were measured to examine the effect of these ST-PGPR on plant growth. In contrast to control (normal soil) conditions, plants that are treated with the GKP KS2_7 and MBD 133 strains did not suffer NaCl-induced salinity stress, thereby resulting in improved growth (Figure 4a–c).

#### 2.7.2. Photosynthetic Pigments (Chlorophyll a, Chlorophyll b, Total Chlorophyll, and Carotenoids) under Salt Stress Environment

**Figure 5:**
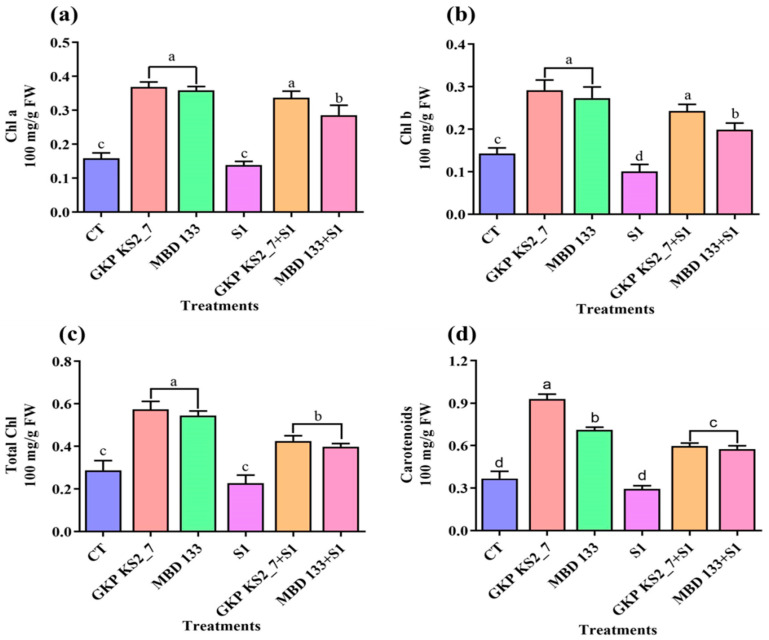
Analyzing the effect of ACCD-producing bacteria on photosynthetic pigments of *P. sativum* under salinity stress. (**a**) Chlorophyll a; (**b**) Chlorophyll b; (**c**) Total chlorophyll contents; and (**d**) Carotenoids contents. Normal soil used as a control: CT—GKP KS2_7 and MBD 133—acdS^+^ bacterial isolates. Salt-stressed: S1—1% NaCl; GKP KS2_7+S1 and MBD 133+S1—PGPR+salt. The values represent the average of three replicates mean ± standard error (SE). Statistical significance is indicated by differences in letters between treatments (One-way ANOVA, Tukey’s multiple range test *p* < 0.05). Similar letters represent no significance, while a different letter is an indication of a significant difference, indicating the significance between them.

The presence of inoculated plants with *P. aeruginosa* GKP KS2_7 and *B. subtilis* MBD 133 considerably increased the levels of photosynthetic pigments (Figure 5a–d). Plants inoculated with GKP KS2_7 strain under NaCl-induced stress conditions produced higher levels of photosynthetic pigments than non-inoculated plants. However, as compared to the control, 1% saline stress caused a reduction in photosynthetic pigments (Figure 5a–d).

#### 2.7.3. Changes in Flavonoids, Phenol, and Proline Contents

**Figure 6:**
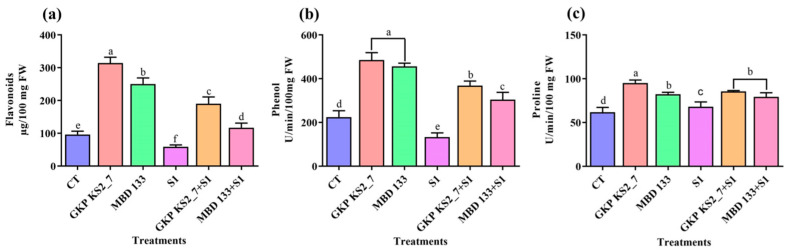
Effect of potent ACCD strains on non-enzymatic antioxidant activities in *P. sativum* under salinity stress. (**a**) Flavonoids content; (**b**) Phenols content; and (**c**) Proline contents. CT—Control (unbacterized seeds); plants inoculated with GKP KS2_7 and MBD 133—ACCD producing bacterial isolates; S1—1% NaCl concentration; GKP KS2_7+S1 and MBD 133+S1—Bacteria+NaCl. Values are the mean of three replicates mean ± standard error (SE). Statistical significance is indicated by differences in letters between treatments (One-way ANOVA, Tukey’s multiple range test *p* < 0.05). Similar letters represent no significance, while a different letter is indicative of a significant difference indicating the significance between them.

The pea plants inoculated with GKP KS2_7 and MBD 133 acdS^+^ isolates promoted the antioxidants level such as flavonoids, phenols, and proline as compared to non-inoculated plants. In comparison to the control, NaCl significantly decreased flavonoids or phenols contents (Figure 6a,b). However, the flavonoids, phenols, and proline content have also been enhanced on inoculation of acdS^+^ isolates when compared to the control. Phenol and proline are two other biomarkers of salt-stressed plants. This means that increased phenolic and proline accumulations are absolutely essential to maintain the osmotic potential of plants and thereby protect them against salinity stress during the growing process. Plant osmolytes were impacted directly by the inoculation of acdS^+^ bacterial isolates (Figure 6a–c). The results further indicated that both the acdS^+^ bacterial strains protect the plants against oxidative damage caused due to induced NaCl-salt stress.

#### 2.7.4. Antioxidants Enzymatic Activities

**Figure 7:**
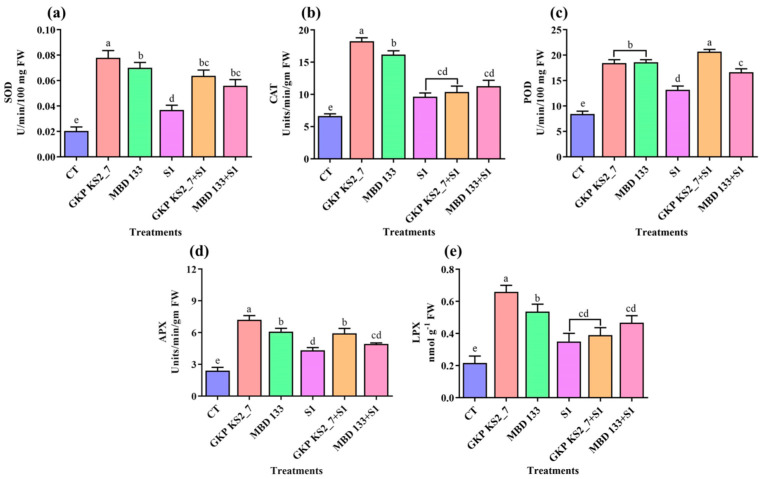
Antioxidant enzyme activities in pea leaves with or without salt and bacterial inoculation. (**a**) superoxide dismutase (SOD) activity; (**b**) Catalase (CAT) activity; (**c**) Peroxidases (POD) activity; and (**d**) Ascorbate peroxidase (APX) activity; (**e**) Lipid peroxidase (LPX) activity. Positive CT—Control without bacterial treatment; plants inoculated with GKP KS2_7 and MBD 133—acdS^+^ bacterial isolates; Negative control: S1—1% NaCl concentration; GKP KS2_7+S1 and MBD 133 + S1 – PGPR + NaCl. Data are expressed as a mean of three replicates mean ± standard error (SE). Statistical significance is indicated by differences in letters between treatments (One-way ANOVA, Tukey’s multiple range test *p* < 0.05). Similar letters represent no significance, while a different letter is indicative of a significant difference indicating the significance between them.

A leaf sample was assessed for antioxidant enzymatic activity, including SOD, CAT, POD, APX, and LPX. In normal and stressed soil conditions, both the acdS^+^ bacterial isolates increased the antioxidant enzymatic activities (Figure 7a–e). The SOD and POD activity was increased by GKP KS2_7 followed by MBD 133 under normal and salinity-induced environments, whereas the MBD 133 strain increases the POD activity under salinity stress (Figure 7d). The APX content was also found to be elevated in GKP KS2_7 inoculated pea plants in normal as well as under saline-stressed soil (Figure 7d).

Catalase and lipid peroxidase activity was increased in acdS^+^ inoculated plants under normal conditions, while under salt stress conditions, bacterial strain MBD 133 significantly increased the CAT and LPX content (Figure 7b,e). The antioxidant enzymatic activity in pea plants was significantly enhanced under saline stress conditions, proving that all these enzymes are protective against harmful oxidative stress.

### 2.8. Correlation Analysis

**Figure 8:**
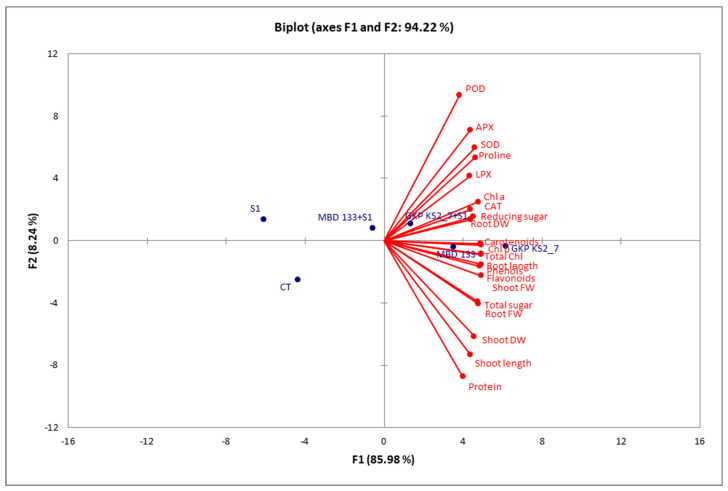
The Pearson correlation biplot analysis between different treatments in *P. sativum*, morphological, and biochemical parameters as well as antioxidants enzymes. Blue colored dots represent the correlation between experimental treatments, and red dots represent the correlation between experimental variables.

The bi-plots correlation analysis revealed that the *P. aeruginosa* GKP KS2_7 strain showed a positive correlation between different variables, such as the effect on morphological, biochemical as well as enzymatic parameters in *P. sativum* under salinity stress conditions (Figure 8). The correlation between traits is indicated by red and blue dots, which represent the correlation between treatments. There was a very strong and positive correlation between variables in the same quadrant that were extremely close together. The combined correlation bi-plot between F1 and F2 revealed a 94.22% variation in which F1 contributed 85.98% and 08.24% for F2. Several significant positive correlations (alpha = 0.05) were found between root and shoot length; root and shoot fresh and dry weight; chlorophylls and carotenoids; TSS, RS, and protein contents; as well as antioxidants enzymes such as POD, APX, SOD, proline, LPX, CAT, phenols, and flavonoids (Figure 8). On the contrary, CT, S1, and MBD 133+S1 were negatively correlated with all other variables. A significant increase in these attributes is directly correlated with an increase in plant biomass yield (Figure 8).

## 3. Discussion

Land salinization is an increasing problem all over the world. A progressive rise in salt content in irrigated agricultural soils is one of the biggest hazards to crop output [33] and has serious consequences for plant growth, resulting in dramatic yield loss [32]. Possibly, this is due to the damaging effects of salinity on the structure of the cell wall [34], increase in ethylene concentration, and hence inhibits root development [35]. A salinity concentration negatively affects seed germination, retarding growth, germination, and other parameters [36]. Many other researchers have shown that the effect of salinity was visible in all the growth parameters, including seed germination, germination rate and time, seedlings, seedling VI, and dry and fresh biomass [16,36,37,38]. The effects of salinity on shoots compared with roots have also been reported by Ramoliya et al. [39], Munns [40], and Orhan [41]. Thus, the present study investigated screening of the ST ACCD-producing strains (GKP KS2_7 and MBD133) in order to determine their growth-promoting characteristics and salinity amelioration effects by reducing stress ethylene and modulating ROS-related enzymes.

These bacteria were previously characterized by Gupta et al. [12] and exert a broad range of plant-beneficial activities such as N_2_ fixation, the production of IAA, P-solubilization, siderophore, ammonia, EPS production, and ACC deaminase activity. The uptake of phosphate, which is greatly reduced under salinity stress, limits plant growth [42]. Under such conditions, ACCD-positive isolates have been shown to improve P uptake by releasing various mineral-dissolving compounds such as protons, CO_2_, hydroxyl ions, and extracellular enzymes, among others [43]. Additionally, the selected strain GKP KS2_7 and MBD 133 produces copious quantities of IAA (Table 1). According to Vimal et al. [44], IAA generated by bacterial strains directly promotes root development by inducing cell elongation and/or acting in response to cell division. IAA production is associated with root growth and structural modifications to respond to stressful situations [45]. In addition to stimulating the elongation of the roots, IAA also stimulates the activity of ACC synthase, an enzyme involved in ACC synthesis. An increase in ACC production would, however, benefit the bacterium since it uses it as a nitrogen (N) source [46]. Both the acdS^+^ strains GKP KS2_7 and MBD133 are capable of producing siderophores that chelate ferric iron and make it available for plant uptake (Table 1). Similar studies have also been reported by Arora et al. [47] and Maheshwari et al. [48]. A major function of the EPS produced by the PGPR is to bind excess Na^+^, thus reducing the concentration of effective salts in the environment. Therefore, this has an indirect influence on alleviating salinity stress [17,49,50,51]. These bacterial strains (GKP KS2_7 and MBD133) also produce a significant amount of ACCD activity at 24 h and have the ability to consume ACC—(Table 2). The ninhydrin test of ACC, an α-amino acid with a cyclopropane ring, allows for the assessment of ACC bacterial consumption. Our findings were in-line with those of Blaha et al. [52] and Onofre-Lemus et al. [53], who reported that a majority of ACC-consuming bacterial isolates come from *Pseudomonas* and *Bacillus* genera. It is absolutely necessary to demonstrate ACCD activity in order to determine if an isolate possesses ACC deaminase, even though growth on minimal media containing ACC as the sole N source is not sufficient. Additionally, the ACCD activity was analyzed using the 2,4-dinitrophenylhydrazine (DNPH) assay to precipitate aldehydes and ketones from carbonyl compounds and measuring the presence of α-ketobutyrate (a by-product of ACC) (Figure 1a) and pyruvate. The results of the current study confirm the findings of previous publications, reporting that salt stress can be counteracted by ACCD-positive bacteria [12,54,55,56]. The FTIR findings showed that ACC deaminase metabolized the ACC. The results were demonstrated based on peak to determine the α-ketobutyrate and ammonia at distinct wavenumbers (Figure 1b). Plants with PGPR that produce ACCD can resist the detrimental effects of stress-induced ethylene produced under salinity stress conditions.

In a prior investigation, we also used PCR to identify the acdS gene at the molecular level and to demonstrate its ACCD activity [12]. The amplified product was subsequently sequenced and compared with the acdS^+^ gene sequences available in the NCBI database (data not shown), revealing 97–98% similarity. The acdS gene is not just found in the plant-beneficial taxa, but it is found in a variety of harmful Proteobacteria [57]. The presence of acdS (and occasionally ACC deaminase activity) in true or opportunistic infections is surprising because ACC deaminase activity is a characteristic that benefits plants. This raises the question of its role in pathogenesis, which may be especially relevant when dealing with phytopathogens. Additionally, it challenges the role this trait may play in ensuring the survival of human pathogens that are true and opportunistic in terrestrial and non-terrestrial environments since ACC deaminase activity may be crucial to nitrogen nutrition in microbial habitats with ACC [58,59]. For instance, *Burkholderiacepacia* genomovar III strains have been discovered in the maize rhizosphere [60]. However, it is unknown if the same strain is capable of colonizing both the rhizosphere and cystic fibrosis patients. The importance of acdS and/or ACC deaminase activity for survival in extraterrestrial habitats is not immediately apparent since, as far as we are aware, ACC has not been observed in hosts that are either animals or human hosts. Within acdS group II, a single *Burkholderia* cluster included every opportunistic human pathogen. It will be necessary to corroborate this observation using other sequences. However, when the acdS sequence was examined, it seemed that there was no direct connection between the ecological strategy of the bacteria and the sequence, as each acdS group contained both harmful and helpful bacteria for plants. As a result, acdS alleles cannot be used as molecular markers to differentiate harmful from non-pathogenic bacteria.

Nevertheless, it should be noted here that the GKP KS2_7 and MBD 133 strains are able to tolerate a salt concentration of about 3%, which makes them ideal for use with salt-stressed soil (Figure 2). ACCD-positive strain inoculation also increases the G%, GI, MGT, TGP, CVG, GRI, and VI, morphological parameters such as root-shoot length, fresh and dry weight of roots and shoots (Table 3). An increase in seedling growth and germination percentage has also been reported by Din et al. [61]. The PGP traits involved in the host plant can be positively correlated to the increase in overall performance upon inoculation with GKP KS2_7 and MBD 133 strains (Figure 3a,b). An increase in root length, plant height, dry matter, and leaf size with the inoculation of PGPR on pepper seeds under saline soil has also been revealed by Latef and Chaoxing [62]. The phytohormone IAA promotes root growth, increases root elongation and lateral branching of plants [51], increases root hair production [63], enhances the germination process (Table 1 and Table 4) [16], and enhances photosynthetic pigments (Figure 5a–d) as previously reported by Kaya et al. [64] and Dodd and Pérez-Alfocea [65]. The results were further illustrated by the ability of the selected GKP KS2_7 and MBD 133 strains to colonize roots, which was demonstrated by counting colony counts in inoculated roots (data not shown).

Environmental stress can be evaluated using chlorophyll content [66]. Salinity can cause chlorosis, which inhibits photosynthesis. Thus, plant response can be determined by pigment degradation [67]. Data demonstrated that chlorophyll content significantly decreases under 1% induced NaCl concentration (Figure 5). The chloroplast structure might be damaged due to salt effects [68,69]; this may lead to an impaired energy transfer from PSII to PSI [70] and, consequently, lowers chlorophyll synthesis in stressed pea crops. The presence of salinity can also reduce stomatal conductance, which destroys biochemical processes and leads to the degradation of chlorophyll content [12]. It is similar to the findings published by Abdelaal et al. [71] during salinity stress in sweet pepper. By inoculating the crop with acdS^+^ bacterial isolates, the photosynthetic pigments of the host plant (Pea) can also be increased while reducing the xylem equilibrium pressure [72,73]. Additionally, Habib et al. [17] confirmed their previous findings, as well as increased photosynthesis [74]. Plants inoculated with PGPR showed altered nitrogen, potassium, and phosphorus uptake, which are responsible for plant growth promotion [75].

A decrease in plant growth parameters under stressful conditions is attributed to stress-induced ethylene. The acdS^+^ bacterial isolates (GKP KS2_7 and MBD 133) overall improved plant growth parameters, biochemical parameters, antioxidant enzymes status, and maintained stress-induced ethylene levels. Mechanisms of plant growth promoting bacteria mediated drought and salinity stress tolerance in vegetable crops [76] and stress tolerance in plants [16,19,26,77,78] have been described for sustainable agriculture. In addition, a group of researchers has analyzed the mitigation of stress ethylene using ST ACCD-producing PGPRs in other plants, which yielded significant results as well [79].

Biochemical tests such as TSS, RS, protein, chlorophylls, carotenoid, phenol, flavonoid, and proline content were further investigated to evaluate the effect of acdS^+^ PGPR (*P. aeruginosa*) and (*B. subtilis*) on plant growth. In order to assess the quantity of SOD, CAT, POD, APX, and LPX, the antioxidant enzyme activity was also tested. Each assay examined a distinct mechanism for coping with salinity stress, as previously stated. Salinity stress can cause oxidative stress in leaves, which can lead to photo-oxidative damage and photo-inhibition in the leaves [80]. By increasing ROS scavenging enzyme production and enhancing the phenyl-propanoid pathway under NaCl stress, flavonoids and phenols were increased, revealing that they play a protective role in plants. Plants with high flavonoid levels also lower the hydroxyl radicals in their cells that are produced upon abiotic (saline) stress. NaCl stress is associated with a change in osmotic pressure inside plant cells [81] and has been shown to have higher proline concentrations in certain plant species, including tomatoes [82]. Inoculation of the selected acdS+ isolates GKP KS2_7 (*P. aeruginosa*) and MBD 133 (*B. subtilis*) revealed an increased level of proline in pea plants (Figure 6c). Under saline circumstances, similar outcomes were reported by Beltrano et al. [83], Islam et al. [84], and Hahm et al. [85] in pepper and mungbean plants.

Plants have evolved antioxidant mechanisms to counter the effects of oxidative stress, including enzymes such as SOD, CAT, POD, APX, and LPX [86]. ACC deaminase-positive bacterial inoculation also prevented the oxidative burst by enhancing antioxidant machinery [12,87]. Our findings also showed that the inoculation of acdS^+^ bacterial strains (GKP KS2_7 and MBD 133) under salt stress resulted in a significant increase in antioxidant activities (Figure 6 and Figure 7). POD plays a crucial role in removing H_2_O_2_ from seedling tissues and reducing oxidative stress [88]. SOD is the first line of defense against ROS, as per Elkelish et al. [89], and it also suppresses the generation of OH radicals, resulting in decreased lipid peroxidation in cellular membranes [90]. In this regard, PGPRs that have ACCD activity is especially important because these bacteria use ACC as a substrate for their growth by modulating the ethylene production during stress [12,16,26,91].

This is the first study to our knowledge to examine the effect of these acdS^+^ isolates on plant-microbe interactions under salinity stress conditions. Evidence from several studies suggests that inoculation of ACCD-positive PGPR can alleviate salinity stress in crops [12,35,72,92,93].

## 4. Materials and Methods

### 4.1. Bacterial Strains and In Vitro Characterization of Bacterial Isolates for Plant Growth-Promoting (PGP) Traits

*Pseudomonas aeruginosa* (GKP KS2_7) and *Bacillus subtilis* (MBD 133) strains were previously isolated from rhizospheric soil [12] based upon their ability to utilize ACC as the sole source of N, present in sterile DF minimal salt medium [94] were used in this study. These isolates tested positive for various PGP activities such as IAA production, P-solubilization, siderophore production, NH_3_ production, EPS production, and ACC deaminase activity. These isolates were grown on either solid (Luria Agar-LA) or liquid (Luria broth-LB) medium (Hi-media Laboratories, Mumbai, India) at 28 ± 2 °C.

Gordon and Weber [95] briefly described the detection of IAA as follows: A supernatant of the LB culture was treated with Salkowski reagent with and without the addition of L-tryptophan (0.22 mg mL^−1^). Supernatants were diluted with Salkowski reagent by a factor of 2:1. After 25 min of incubation at room temperature, OD_530_ nm was determined.

The amount of phosphate that the chosen isolates solubilized was measured according to the procedure of Nautiyal [96]. Briefly, test tubes containing 5 mL of NBRI-BPB (National Botanical Research Institute’s phosphate medium) plates containing (bromophenol blue dye) medium and 50 µL of overnight-grown bacterial culture, were inoculated. The test tubes were then incubated at 28 ± 2 °C for 48 h while being shaken at 180 rpm. After centrifuging the cells at 10,000 rpm for 10 min, the supernatant was used to spectrophotometrically measure the quantity of solubilized phosphate at 490 nm.

The phenol-sulphuric acid technique was used to quantify EPS production [97]. A 10 mL overnight-grown old culture of bacterial isolates with equivalent amounts of phenol (0.5 M) and 1 mL sulphuric acid (9.8 M) were utilized in order to measure the EPS generated at an absorbance of 490 nm.

By using the chrome azurol sulfonate (CAS)-shuttle assay, the strain siderophore production was quantitatively evaluated. For this test, the strains were cultivated on succinate medium [98] and incubated for 24–48 h at 28 °C with continual shaking at 120 rpm on a rotating incubator. After incubation, the fermented broth was centrifuged at 12,000 rpm for 15 min, and 0.5 mL of the cell-free supernatant was combined with 0.5 mL of the CAS reagent. The absorbance at 630 nm was then measured in comparison to the control made up of 0.5 mL of un-inoculated broth and 0.5 mL of the CAS reagent. The following formula was used to determine the siderophore content in an aliquot [99]:

$$\%\;\mathrm{Siderophore}\;\mathrm{Units}=\frac{Ar-As}{Ar}100$$

where,

- *Ar* = sample absorbance at 630 nm (un-inoculated medium + CAS assay solution)
- *As* = sample absorbance at 630 nm (supernatant + CAS assay solution)

For NH_3_ production, bacterial isolates were examined for their ability to produce ammonia in peptone water. Freshly developed cultures were added to 10 mL of peptone water in each test tube before being incubated for 48–72 h at 28 ± 2 °C. Each tube received 0.5 mL of Nessler’s reagent. The transformation of brown to yellow color was a positive sign for ammonia production [100].

### 4.2. ACC Consumption Assay Using Colorimetric Ninhydrin Assay

A 500 mg of ninhydrin (Sigma-Aldrich, Gillingham, UK) and 15 mg of ascorbic acid (AA) in 60 mL of ethylene glycol (Hi-media Laboratories, Mumbai, India) were then mixed and stored at −20 °C. The reagent was then mixed with 60 mL of 1 molar citrate buffer (pH 6.0) before use. Here, ethylene glycol was utilized as a solvent to sustain the ninhydrin reagent and color development. To obtain the respective ACC working concentrations of 0.005, 0.01, 0.015, 0.02, 0.03, 0.04, 0.05, 0.10, 0.15, 0.20, 0.25, 0.30, 0.40 and 0.50 mmol^−1^), the DF-ACC medium (with a concentration of 5.0 mmol^−1^ ACC) further was diluted. A glass test tube was capped and shaken, then dipped in a boiling water bath after adding 1 mL of ACC working solution and 2 mL of Ninhydrin reagent. After 15 min, the tubes were placed into the H_2_O bath for 2 min at 37 °C before being shaken for 30 s [101,102]. The sample solution was allowed to cool at room temperature for 10 min, then transferred to the cuvette, and measured the absorbance at 570 nm using a UV-visible spectrophotometer (Eppendorf UV-VIS Kinetic Biospectrometer, Hamurg, Germany). As a blank, the DF-medium was used. Each working solution was assayed in triplicate. In this experiment, a linear calibration curve was obtained by using standard Ninhydrin assays on ACC solutions ranging from 0.015 to 0.5 mmol^−1^ after the reaction, and this result is significantly correlated (R^2^ = 0.999). It took twice as long for Ruhemann’s purple to develop fully in plate wells as in test tubes. For each ACC concentration used in the PCR-plate assay, the absorbance value at 570 nm was lower than that of the standard assay. Furthermore, ACC was determined by ninhydrin assay using 1 mL of a 10-fold diluted supernatant of bacteria culture. Following an incubation period in the DF-ACC medium, the ACC concentrations in a given bacterial culture were identical in both assays at the 0.5% confidence level.

### 4.3. Quantification and Confirmation of ACCD Activity by Fourier Transform Infrared (FTIR) Spectra Analysis

The enzymatic activity of identified ST-bacterial strains was qualitatively assessed using both agar plate and in-broth culture methods. The ACCD production was estimated based on the amount of α-ketobutyrate released via its hydrolysis [55]. For this, a pellet of an overnight grown bacterial culture was harvested and washed with Tris-HCl buffer (pH 7.6) in DF salts minimal medium [94] with 5 mM ACC as the N-source. After the collection of cell pellets, toluene was used to treat the pellets, and 20 µL (0.5 M ACC) was mixed with 200 µL of tolunized cells and incubated at 28 ± 2 °C for 15 min. In order to create the final reaction mixture, 1 mL of 0.56 M HCl and 300 µL of DNPH were added to 1 mL of ACC-treated tolunized cells and incubated at 28 ± 2 °C for 30 min. Thereafter, 2 mL of 2 N NaOH was added to the final reaction mixture to develop purple color production for visual detection, and O.D. was measured at 540 nm in a UV-visible spectrophotometer (Eppendorf UV-VIS Kinetic Biospectrometer). The quantity of α-ketobutyrate generated per mg of protein per h served as an indicator of the ACC deaminase enzyme’s catalytic activity. A reference curve of α-ketobutyrate (Sigma-Aldrich, St. Louis, MI, USA) was constructed to calibrate the mean value [55,103].

The amount of α-ketobutyrate released by the ACCD is measured in nanomoles per milligram of cell protein per h. In addition, Fourier-transform infrared spectroscopy (FTIR) (Perkin Elmer Spectrum Version, 10.03.06 instrument, Waltham, MA, USA) was used to analyze α-ketobutyrate liberation from ACC deamination in DF minimal salt media by the isolates with maximum ACCD activity using potassium bromide (KBr) cell pellet. An FTIR analysis of ACC spectra was conducted to confirm the release of α-ketobutyrate. The test was conducted by incubating 200 µL of tolunized bacterial cells as previously described by Penrose and Glick [55] with 0.5 M ACC at 30 °C for 30 min, followed by centrifugation at 12,000 rpm for 5 min at room temperature. Purified reaction mixtures were lyophilized and mixed in a 3:1 ratio with KBr to make pellets, and the pellets were used for FTIR spectra analysis. Analyses of the experimental IR data were compared to those of standard FTIR analysis of α-ketobutyrate [32].

### 4.4. Assessment of Maximum Salt Tolerance Level (MTL) of acdS^+^ Bacterial Isolates

Assays were conducted on bacterial isolates to assess their susceptibility to salinity (NaCl) stress using NB and varying NaCl (Hi-media Laboratories, Mumbai, India) concentrations (1-, 2-, 3- and 5% NaCl *w/v*) at 600 nm over a range of times [18,104].

### 4.5. Investigation of PGP Activity of PGPR and Seedlings Parameters in Pisum sativum under Induced NaCl Stress Condition

The impact of these bacterial isolates on pea seeds under NaCl stress during germination was subsequently tested using in vitro growth-promoting assays. In order to prepare bacterial inoculum, overnight grown bacterial cells with 1 × 10^8^ CFU mL^−1^ were centrifuged at 6000 rpm for 10 min and rinsed twice with 50 mM phosphate saline buffer (PSB) solution and re-suspended thereof in a ratio of 1:1 in double-distilled water (ddw) to maintain the uniform cell density of 1 × 10^8^ CFU mL^−1^. Surface sterilization of pea seed was performed with 70% ethanol (*v/v*) for 1 min, and then in 0.1% mercuric chloride (HgCl_2_) purchased from (Sigma Chemical Co., Mumbai, India) (≥99.5%) and used for pot trial assays.

The seed germination rates were measured regularly until the 10th day after sowing, as soon as they germinated. Following the International Seed Testing Association (ISTA) method, the germination percentage (G%) was calculated as described by Gupta et al. [16] and Matthews et al. [105].

$$G\%=\frac{\mathrm{number}\;\mathrm{of}\;\mathrm{normally}\;\mathrm{germinated}\;\mathrm{seeds}}{\mathrm{total}\;\mathrm{number}\;\mathrm{of}\;\mathrm{seeds}\;\mathrm{sown}}\times 100$$

The following formula was used to calculate the germination index (GI):

$$\mathrm{GI}=\Sigma \frac{\mathrm{Gt}}{\mathrm{Tt}}$$

where Gt is the number of seeds germinated on t day, and Tt is the number of days. Other growth parameters such as mean germination time (MGT), germination rate index (GRI), total germination (TG)%, vigor index (VI), and coefficient of the velocity of germination (CVG) were also measured [16,106].

### 4.6. Collection of Soil Sample

The soil sample was collected from an area near Kumarganj, Ayodhya, Uttar Pradesh, India (26.5468° N, 81.8402° E). The pH of the soil was tested using a pH strip and was found to be ~8.5. Polyethylene bags were used to transport the samples to the laboratory.

### 4.7. Greenhouse Experiment

#### 4.7.1. Seed Sterilization and Bacterization

The *P. sativum* seeds were used for growth-promoting experiments in soil conditions. Following surface sterilization with 70% ethanol (*v/v*) for 1 min and then in 0.1% HgCl_2_ solution (*w/v*) for 10 min before being rinsed with sterile deionized H_2_O (four to five times) [107]. After being sterilized and inoculated with appropriate bacterial suspensions, the pea seeds were air-dried aseptically in the laminar airflow for 1 h. During the control study, sterile H_2_O was used to immerse surface sterilized, unprimed pea seeds.

#### 4.7.2. Pot Experiment

Sterile thermo-coal pots with the sizes of 5.95 cm × 5.3 cm × 4.2 cm the seeds were sowed in the tyndallized autoclaved soil. For each treatment in normal soil and salt-amended soil, there were 5 replicates control (normal soil), bacteria-inoculated (GKP KS2_7 and MBD 133), salt (1% NaCl), and bacteria + salt. The soil was artificially stressed by adding 1% NaCl to it. A randomized block design was used in the pot experimental trials for pea growth. During the growing process, the pots were maintained in GH conditions and irrigated with tap water regularly. Plants were monitored regularly and harvested after 25 days of sowing (DAS) to evaluate the morphological characteristics, including lengths, and fresh and dry weight of shoot-root. Additionally, biochemical changes in harvested plants were also examined under various treatment conditions.

### 4.8. Analysis of Physiological and Morphological Characteristics of Pisum sativum

After 25 days of growth under saline or non-saline conditions, root/shoot length, root/shoot fresh and dry weight, and other growth-related parameters were measured after pea plants were harvested (three plants per treatment). In order to determine the dry biomass of pea plants, the roots and shoots were ovens-dried separately for 3 days at 60 ℃ and then weighed.

#### 4.8.1. Biochemical Analysis

##### Estimation of Total Soluble Sugar (TSS), Reducing Sugar (RS), and Protein

Using the method developed by Dubois et al. [108], the TSS content was determined. RSs were estimated by following Somogy’s method as amended by Nelson [109] by using glucose as a standard. Using the Lowry et al. [110] method, the estimation of proteins was performed by taking bovine serum albumin (BSA) as standard (Hi-media Laboratories, Mumbai, India).

##### Photosynthetic Pigments

The 100 mg of each treatment fresh leaves were homogenized in 80% acetone in a mortar and pestle that had been pre-chilled. The number of chlorophylls and carotenoids in the leaves was calculated using the following formula [111,112].

Chlorophyll a (mg/g FW) = (12.7 × A_663_) − (2.69 × A_645_) × V

1000 × W × a

Chlorophyll b (mg/g FW) = (32.9 × A_645_) − (4.88 × A_663_) × V

1000 × W × a

Total Chlorophyll = Chlorophyll a + Chlorophyll b = (20.2 × A_648_) − (8.02 × A_664_) × V

1000 × W × a

Carotenoids (mg/g FW) = A_480_ + (0.114 × A_663_) − (0.638 × A_645_)

where,

- A—Absorbance at a specific wavelength (nm)
- W—Fresh weight of the sample (g)
- V—Volume of the 100% ethanol (mL)
- a—Length of the light path in the cell (1 cm)

#### 4.8.2. Antioxidants Enzymes

##### Non-Enzymatic Assays—Phenols, Flavonoids, and Proline

By using the Folin-Ciocalteu methodology, McDonald et al. [113] evaluated the total phenolic content in plant samples using Gallic acid (Hi-media Laboratories, Mumbai, India) as a standard. The quantitative estimate was conducted using spectrophotometric analysis based on complex flavonoid-aluminum formation through the Aluminum chloride method of Chang et al. [114], using Quercetin (Hi-media Laboratories, Mumbai, India) as the standard. The proline estimation was performed based on the methodology proposed by Bates et al. [115]. The concentration was calculated by comparing it to the L-proline standard curve (Sigma-Aldrich, USA).

##### Enzymatic Assay—Superoxide Dismutase (SOD), Catalase (CAT), Peroxidase (POD), Ascorbate Peroxidase (APX), and Lipid Peroxidase (LPX)

In a pre-chilled mortar and pestle, 100 mg of fresh *P. sativum* leaves were homogenized in 2 mL of ice-chilled 50 mM phosphate buffer (pH 8.0). By centrifuging the homogenate for 12 min at 4 °C at 12,000 rpm, the antioxidant enzymes were measured.

The SOD estimate was calculated using the Kono [116] methodology. The approach is based on the principle that superoxide radicals are generated through the auto-oxidation of hydroxylamine hydrochloride, preventing the reduction in nitroblue tetrazolium (NBT) dye by SOD. The absorbance was measured in a UV Spectrophotometer (Eppendorf UV-VIS Kinetic Biospectrometer) at 560 nm.

The activity of catalase was determined using the Aebi [117] method that catalyzes the conversion of H_2_O_2_ to form H_2_O and O_2_. CAT activity was monitored by observing the disappearance of H_2_O_2_ at 240 nm after adding enzyme extract to the reaction mixture. The extinction coefficient (3.99 mm^−1^·cm^−1^) was used to quantify the activity.

The peroxidase assay was accomplished by Chance and Maehly [118] and quantified by the extinction coefficient (2.13 mm^−1^·cm^−1^). POX activity was quantified by measuring mmol H_2_O_2_ decomposed mL^−1^min^−1^. The quantity of enzyme necessary to break-down one mole of H_2_O_2_ per min at 25 °C was defined as an enzyme unit.

The APX activity of leaves was assessed at 290 nm using the methodology given by Chen and Asada [119]. As the ascorbate oxidized, the absorbance decreased at 290 nm (absorbance coefficient 2.8 mM^−1^·cm^−1^), indicating the activity of APX.

As per the method prescribed by Ayala et al. [120], lipid peroxidation can easily be detected by measuring the level of malonyl di-aldehyde (MDA), a by-product of the process.

### 4.9. Statistical Analysis

A minimum of three biological replicates were used for the experiments, and each data point shown in the results was the meaning of these replicates. A mean ± standard error is represented by the error bars (mean ± SE). Statistical evaluation of two groups of means was compared by unpaired *t*-test and two-tailed *p* value. In contrast, all the means were performed by using One-way ANOVA analysis followed by Two-way ANOVA analysis, and statistically meaningful data were compared using “Tukey’s Multiple Comparison Test” performed by using GraphPad Software (GraphPad Prism 8.00, San Diego, CA, USA). Graphs with different letters demonstrate significant changes between treatments (*p* < 0.05), whereas graphs with the same values indicate the non-significant differences between samples. An analysis of bi-plots correlation was conducted using XL-STAT 2014.5.03 by plotting the mean values of all variables.

## 5. Conclusions

In the present study, we characterized two potential strains, GKP KS2_7 and MBD 133 (*P. aeruginosa* and *B. subtilis*), that functions as ST ACCD-producing PGPR. These strains exhibit important PGP traits, such as IAA production, P-solubilization, NH_3_ production, EPS production, siderophore production, and ACCD activity, which are necessary for plant development enhancement, especially in the presence of salinity stress. The ninhydrin-ACC test is a quick and effective way to screen bacteria that have ACC deaminase from a large number of bacterial isolates for ACC consumption. The results indicated that these bacteria have the ability for ACC consumption and hence possess ACC deaminase activity. Further confirmation of their ACCD activity was provided by FTIR analysis. The inoculation of *B. subtilis* and *P. aeruginosa,* as described in this work, is found to protect the pea seedlings under salinity stress, thereby decreasing stress ethylene, ROS and increasing the entire seedling, morphological as well as biochemical parameters under NaCl stress conditions for plant growth promotion. Therefore, this study provides important insights into coping with salinity stress using PGPR as a biofertilizer, which may replace the use of chemical fertilizers.

Hence, the use of these strains can boost the growth of Pea plants and other agro-economically important crop plants by eliminating ion imbalances, suppressing oxidative stress levels, and increasing the levels of osmoprotectants and antioxidants. The findings of this study will aid in the development of salt stress management solutions for major crops grown in challenging or stressed areas.

## Figures and Tables

**Table 1:** Characteristics of PGPR *Pseudomonas aeruginosa* (GKP KS2_7) and *Bacillus subtilis* (MBD 133).

Bacterial Strains	IAA (with Tryptophan)	P-Solubilization	Siderophore Production	NH_3_ Production	EPS Production	ACC Deaminase Production	NCBI Gene Accession No.
	(µg mL^−1^)	(µg mL^−1^)	(%)				
*Pseudomonas* *aeruginosa* (GKP KS2_7)	84.6 ± 2.16 ^a^	113 ± 2.4 ^a^	28.7 ± 1.3 ^a^	+	+	+	KT429595.1
*Bacillus subtilis* (MBD 133)	63.4± 1.73 ^b^	86 ± 2.1 ^b^	20.1 ± 0.7 ^b^	+	+	+	KT429586.1

(+)—Positive production; IAA—Indole acetic acid; P—Phosphate; NH_3_—Ammonia; EPS—Exopolysaccharide; ACCD—1-aminocyclopropane-1-carboxylic acid deaminase. Data are average values of three replicates mean ± standard error (SE). Values are the mean of three replicates. Statistical significance is indicated by differences in letters between treatments (Two-way ANOVA, Tukey’s multiple range test *p* < 0.05). Similar letters represent no significance, while a different letter is an indication of a significant difference, indicating the significance between them.

**Table 2:** ACC deaminase consumption by colorimetric Ninhydrin-ACC assays measured at 0 h and 24 h after bacterial inoculation.

Bacterial Isolates	Genus Affiliation	16 s rRNA Gene Sequence Accession No.	ACC Concentration (mmol l^−1^)	
			0 h	24 h
GKP KS2_7	*Pseudomonas aeruginosa*	KT429595.1	0.597 ± 0.002 ^a^	0.3411 ± 0.0016 ^c^
MBD 133	*Bacillus subtilis*	KT429586.1	0.220 ± 0.002 ^b^	0.0301 ± 0.0011 ^d^

Data are average values of three replicates mean ± standard error (SE). Values are the mean of three replicates. Statistical significance is indicated by differences in letters between treatments (Two-way ANOVA, Tukey’s multiple comparison test *p* < 0.05). A different letter is an indication of a significant difference, indicating the significance between them.

**Table 3:** Effect of PGPR on *Pisum sativum* seedling growth parameters grown in 1% NaCl concentration compared to non-inoculated control seedlings.

Treatments	Germination Percentage (G%)	Germination Index (GI)	Mean Germination Time (MGT)	Total Germination Percentage (TGP)	Coefficient of Velocity of Germination(CVG)	Germination Rate Index (GRI)	Vigour Index (VI)
CT	58 ± 2.60 ^c^	176.67 ± 2.97 ^b^	4.17 ± 0.14 ^c^	24 ± 2.31 ^c^	6.6 ± 0.29 ^c^	1693.26 ± 283.45 ^c^	1221.09 ± 216.51 ^b^
GKP KS2_7	91 ± 2.60 ^a^	316.67 ± 9.38 ^a^	7.96 ± 0.25 ^a^	52 ± 2.31 ^a^	18 ± 2.89 ^a^	2908.73 ± 230.93 ^a^	3284.39 ± 173.28 ^a^
MBD 133	67 ± 2.03 ^b^	233.33 ± 7.13 ^b^	5.65 ± 0.32 ^b^	36 ± 3.64 ^b^	9.36 ± 2.02 ^b^	2087.32 ± 173.26 ^b^	3201.02 ± 230.94 ^a^
S1	18 ± 1.45 ^e^	81.33 ± 4.90 ^d^	1.47 ± 0.23 ^d^	14 ± 3.62 ^d^	1.1 ± 0.98 ^c^	523.65 ± 101.04 ^d^	350.29 ± 28.87 ^c^
GKP KS2_7+S1	39 ± 2.31 ^d^	141.67 ± 4.90 ^c^	3.567 ± 0.21 ^c^	28 ± 2.03 ^c^	3.6 ± 0.45 ^c^	1287.71 ± 285.79 ^c^	1459.33 ± 209.11 ^b^
MBD 133+S1	26 ± 1.45 ^d^	116.67 ± 4.84 ^d^	2.32 ± 0.15 ^d^	24 ± 2.31 ^c^	3.01 ± 0.58 ^c^	968.26 ± 248.26 ^c^	1327.56 ± 129.91 ^b^

CT—Control without bacterial inoculation; Bacterial isolates—GKP KS2_7 (*Pseudomonas aeruginosa*) and MBD 133 (*Bacillus subtilis*); S1—1% NaCl. Values are the mean of three replicates. Statistical significance is indicated by differences in letters between treatments (One-way ANOVA, Tukey’s multiple range test *p* < 0.05). Similar letters represent no significance, while a different letter is an indication of a significant difference, indicating the significance between them.

**Table 4:** Effect of acdS^+^ PGPR on pea plant growth parameters after 25 days after inoculation at 1% NaCl treatment compared to non-inoculated control.

Treatments	Fresh Weight (g)		Dry Weight (g)		Length (cm)	
	Root	Shoot	Root	Shoot	Root	Shoot
CT	0.525 ± 0.066 ^c^	0.621 ± 0.025 ^d^	0.059 ± 0.006 ^d^	0.068 ± 0.006 ^bc^	7.033 ± 0.549 ^c^	9.984 ± 0.234 ^c^
GKP KS2_7	1.051 ± 0.081 ^a^	1.088 ± 0.069 ^a^	0.105 ± 0.007 ^a^	0.093 ± 0.003 ^a^	15.123 ± 0.666 ^a^	13.413 ± 0.806 ^a^
MBD 133	0.886 ± 0.051 ^b^	1.011 ± 0.059 ^a^	0.071 ± 0.006 ^bc^	0.085 ± 0.003 ^a^	14.077 ± 1.222 ^a^	11.731 ± 0.455 ^b^
S1	0.244 ± 0.073 ^e^	0.405 ± 0.032 ^e^	0.039 ± 0.003 ^e^	0.040 ± 0.008 ^d^	4.675 ± 0.347 ^d^	5.067 ± 0.296 ^d^
GKP KS2_7+S1	0.745 ± 0.072 ^bc^	0.835 ± 0.025 ^b^	0.091 ± 0.005 ^b^	0.073 ± 0.002 ^b^	11.332 ± 0.882 ^b^	10.013 ± 0.654 ^c^
MBD 133+S1	0.592 ± 0.071 ^c^	0.730 ± 0.038 ^c^	0.079 ± 0.008 ^bc^	0.066 ± 0.002 ^bc^	10.241 ± 0.389 ^b^	9.233 ± 0.498 ^c^

Values are the mean of five replicates mean ± standard error (SE). CT—Control; GKP KS2_7—*Pseudomonas aeruginosa* and MBD 133—*Bacillus subtilis*; Salt-stressed: S1—1% NaCl; GKP KS2_7+S and MBD 133+S—PGPR+salt. Statistical significance is indicated by differences in letters between treatments (One-way ANOVA, Tukey’s multiple range test *p* < 0.05). Similar letters represent no significance, while a different letter is an indication of a significant difference, indicating the significance between them. Data of root and shoot lengths were measured at 25 DAS.

## Data Availability

Not Applicable.
